# Broadband Perfect Absorber Based on TiN-Nanocone Metasurface

**DOI:** 10.3390/nano8070485

**Published:** 2018-07-01

**Authors:** Dewang Huo, Jingwen Zhang, Yingce Wang, Chao Wang, Hang Su, Hua Zhao

**Affiliations:** 1 Institute of Modern Optics, Department of Physics, Harbin Institute of Technology, Harbin 150001, China; dwhuo@sina.com (D.H.); jingwenz@hit.edu.cn (J.Z.); wangyingce_hit@163.com (Y.W.); wangchao_hit@sina.com (C.W.); suhanghit@126.com (H.S.); 2 Key Laboratory of Micro-Optics and Photonics Technology of Heilongjiang Province, Harbin 150001, China

**Keywords:** absorber, metasurface, refractory titanium nitride, thermophotovoltaics

## Abstract

Based on an integrated array of refractory titanium nitride (TiN), a metasurface perfect absorber (MPA) in the visible-to-near infrared (NIR) band is reported. The systematic and detailed simulation study of the absorption of the MPA is performed with the finite-different time-domain (FDTD) method. Tailoring the structure, the MPA realizes as high an average as 99.6% broadband absorption, ranging from 400 nm to 1500 nm. The broadband perfect absorption can be attributed to localized surface plasmonic resonance (LSPR), excited by the continuous diameter evolution from the apex to the base of the nanocone, and the gap plasmons excited among the nanocones, as well as in the spacer layer at longer wavelengths. Particularly, the coupling of the resonances is essentially behind the broadening of the absorption spectrum. We also evaluated the electric field intensity and polarization-dependence of the nanocone MPA to offer further physical insight into light trapping capability. The MPA shows about 90% average absorption even at an oblique incidence up to 50°, which improves the acceptance capability of light-harvesting system applications. This unique design with the TiN nanocone array/aluminium oxide (Al_2_O_3_)/TiN structure shows potential in imminent applications in light trapping and thermophotovoltaics.

## 1. Introduction

The perfect absorber has drawn great attention due to the flexible adjustability in optical designs and its potential application including sensing [1], infrared imaging [2,3], solar energy harvesting [4,5,6], and so on [7,8,9,10,11]. Due to the development of nanofabrication and characterization techniques in the past decade, broadband absorbers based on various nanostructures have been widely investigated [12,13,14]. One promising approach for the light absorption enhancement, is light trapping through nanoscale texturing, such as nanowire [15,16], nanocone [17,18,19], or nanodome [20] structures. These structures have demonstrated significant light absorption improvements for various solar cells. In particular, solar thermophotovoltaic systems demand materials with high temperature stability [21]. Titanium nitride (TiN) [22], with a melting point as high as 2930 °C, high temperature durability, plasmonic resonance in the visible-to-NIR range (and hence plasmonic absorption), is a candidate for thermophotovoltaic systems. The perfect absorption based on the TiN square-ring array was first studied with broadband absorption, throughout the entire visible regime [21]. Then, the absorber based on a TiN nanodisk array was studied with perfect absorption, from 400 nm to 700 nm [23]. Furthermore, with the hexagonal array and aluminum substrate, the absorption of TiN metamaterial absorber was broadened to a wider spectrum range from 400 nm to 850 nm with an average absorption of 98.1% [24]. Yet, the absorption band of the TiN nanodisk metamaterial absorber is not broad enough to cover the solar radiation spectrum as much as possible. Some work needs to be done to further improve the perfect absorption band. As some research with the silicon nanocone matasurface [25,26] suggests, the introduction of the nanocone structure performs better than the nanopillar structure, with a broader absorption band. In this work, the broadband metasurface perfect absorber (MPA) based on TiN nanocone array is studied. The resulting metamaterial absorber exhibits polarization-independent near unit absorption, in the visible-to-NIR region from 400 nm to 1500 nm, which is ready to be applied in the high temperature applications, such as solar thermophotovoltaics.

## 2. Methods

**Figure 1:**
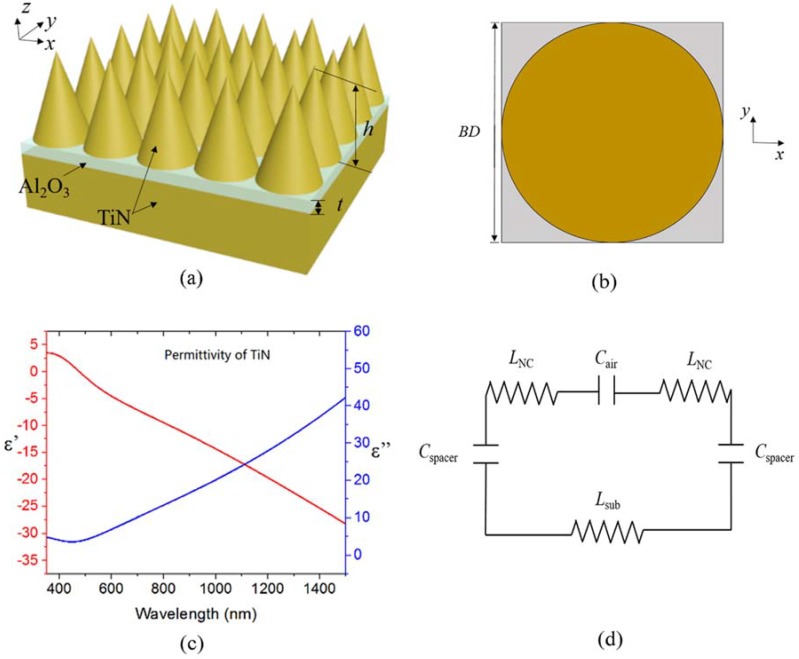
The schematic of the absorber (**a**) and top view of a unit cell (**b**); (**c**) The permittivity curves of TiN; (**d**) LC model of the proposed MPA.

The proposed metamaterial absorber is a three-layer structure as illustrated in Figure 1a. The topmost layer is composed of a TiN nanocone array arranged in a square lattice with a periodicity of *P*. The nanocone is defined with two parameters: height *h* and base diameter *BD* equal to *P*. Al_2_O_3,_ with a melting point of 2000 °C, is introduced as the spacer layer with thickness *t*. We choose TiN as the substrate, with a thickness of 500 nm to prevent transmission of the incident light.

With the commercial software package *LUMERICAL FDTD Solutions*, the finite-difference time-domain (FDTD) method is conducted for plasmonic modeling. The TiN nanocone array is arranged in a square lattice in the *x-y* plane. The incident direction of light with *x*-polarization is normal to the substrate surface along with the backward *z*-direction. In the simulation setup, a square unit cell is chosen to stand for the MPA, as shown in Figure 1b. Then the periodic boundary conditions are set on the x-direction and y-direction. The perfectly matched layer conditions (PML), i.e., absorbing boundary conditions, are set in *z*-direction. The reflection *R* with the intensity of the incident light is detected with a power monitor located behind the radiation source of the plane wave, and the normalized transmission *T* is detected with a power monitor located at the bottom of the substrate. The absorption *A* can be calculated from the corresponding reflectance and transmittance as *A* = 1 − *R* − *T*. The TiN substrate acts as a mirror to form a resonance cavity with the nanocones. In the calculation, nonuniform meshes are employed with a minimum mesh size of 1.0 nm × 1.0 nm × 2.0 nm.

The dispersion curves of TiN shown in Figure 1c, and the corresponding parameters of the spacer layer, are both obtained from the default material list of the software from the *Handbook of Optical Constants of Solids*. [27]. Almost throughout the entire visible-NIR range, the real part of the permittivity of TiN is below zero, which is metal-like enough to support localized surface plasmonic resonance (LSPR), resulting in enhanced resonant absorption. The incident electromagnetic field is able to be efficiently absorbed due to the enhanced field and high imaginary part of permittivity of TiN. Thus, the permittivity is important at each wavelength that the under-zero real part of permittivity causes. The enhanced field and the high imaginary part of permittivity ensures that the energy is efficiently absorbed in the visible-to-NIR range.

To state the possible preparation process of the proposed MPA, the titanium nitride film can be fabricated by the pulsed laser deposition method [28] at room temperature and without using lattice-matched substrate. Additionally, an aluminum film with desired thickness can be fabricated on the TiN substrate using evaporation. Annealing the aluminum film at certain temperatures within an oxygen atmosphere will turn the aluminum film into aluminum oxide film, totally. In order to get stoichiometry, the preparation parameters (such as the oxygen pressure) should be optimized. For the TiN nanocone preparation, the TiN film can be first fabricated onto the alumina film, then, a method in Ref. [29] can be adopted to prepare nanocone as follows. First, a monolayer polystyrene sphere can be self-assembled on the prepared film. Second, oxygen reactive ion etching can be used to etch the monolayer polystyrene sphere. In the etching process, the polystyrene spheres will become smaller gradually, and a nanocone shape will be generated. During this preparation process, the period, height, and size of the nanocone can be easily controlled by the diameter of polystyrene sphere and etching parameters. As for the details of preparation, one can refer to the References [28] and [29].

## 3. Results and Discussion

**Figure 2:**
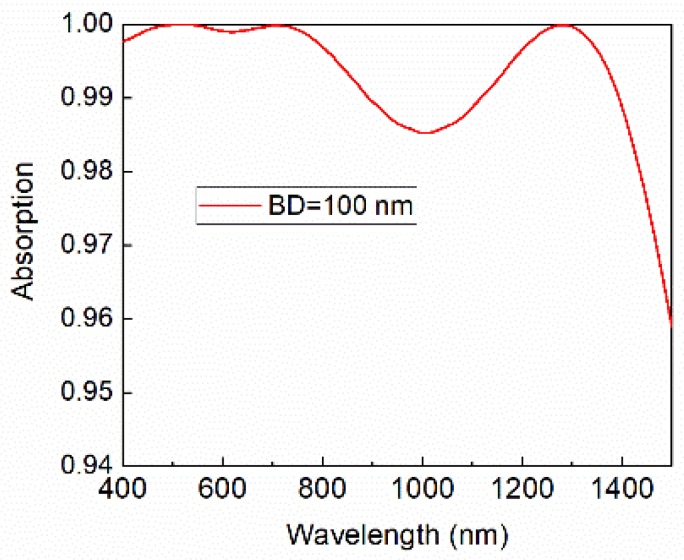
Absorption spectrum of the TiN nanocone MPA.

With parameters of *BD* = 100 nm, *h* = 330 nm and *t* = 25 nm, the MPA with the TiN nanocone array shows great absorption with the average absorbance of 99.6%, from 400 nm to 1500 nm, as shown in Figure 2 (which contains over 90% energy of solar radiation).

**Figure 3:**
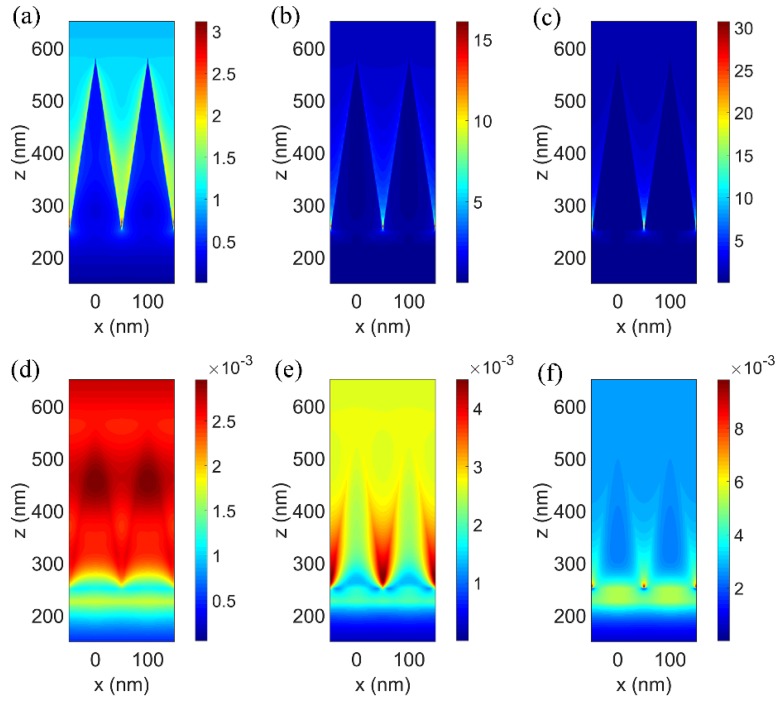
Electric field distributions at wavelengths as (**a**) 400 nm; (**b**) 700 nm; (**c**) 1300 nm and magnetic field distribution at (**d**) 400 nm; (**e**) 700 nm; (**f**) 1300 nm. The units of the electric and magnetic field are V/m and A/m, respectively.

In the literature, the spectral response of a nanoscale resonator made of a high-index dielectric material is dominated by the magnetic and electric resonances that are induced in it by the incident field [30]. The broadband perfect absorption of our proposed TiN nanocone structure is attributed to the excited electric resonance in the shorter wavelength range, magnetic resonance in the longer wavelength range, and their coupling. The electric and magnetic field distributions in the MPA are analyzed as shown in Figure 3. As the diameter of the cross section continuously changes from the base to the apex, light with each wavelength could find an optimum resonance condition in nanocone array [25] as shown in Figure 3a–c. The electric resonance excited by the incident electromagnetic field, which offers a leak channel for the incident light into the structure and reducing reflection as well. The reflectance reduction in the entire visible-to-NIR band is much better than nanodisk structures [24]. At the shorter wavelengths such as 400 nm, the electric resonances are excited in the base corner and the surface of the TiN nanocone, as shown in Figure 3a, so the resonances offer two leak channels: into the TiN nanocone and into the gap. The energy entering into the nanocone from the surface is absorbed due to the large imaginary part of the permittivity and enhanced electromagnetic field. The rest of the incident energy going inside the gap between the nanocone array and substrate is reflected by the substrate and enters the TiN nanocone from the bottom of the nanocone. The electric resonant absorption is dominant at the short wavelength range and the magnetic resonances were barely excited as seen from Figure 3d. As the wavelength is long, such as 700 nm and 1300 nm, the electric resonances can be significantly excited at the base corners of the TiN nanocone as shown in Figure 3b,c, which indicates that the incident energy is mostly leaked into the gaps. At the same time, the magnetic resonance in the gap is dramatically excited. As shown in Figure 3e, the gap plasmon is strongly excited between the adjacent nanocones, which confines the incident energy among the nanocones and permits the TiN to absorb it. When the wavelength is 1300 nm, the magnetic resonance in the spacer layer shows up as shown in Figure 3f, to ensure the energy confines in the spacer layer and enters into the TiN from the bottom of the nanocone. Since the field is significantly enhanced, the energy is perfectly absorbed in the TiN nanocone at the presence of excited resonances. Therefore, the magnetic resonance in the gap plays an even more important role in the perfect absorption phenomena. The broadening of the perfect absorption spectrum stems mainly from the coupling of the electric and magnetic resonances.

The resonance of particle arrays are controlled by the particle’s sizes and the periodicity of the arrays [31]. The absorption spectra, with respect to base diameter and height of the TiN nanocone, and thickness of the Al_2_O_3_ spacer layer, respectively, have been studied with normally incident, *x*-polarized light.

**Figure 4:**
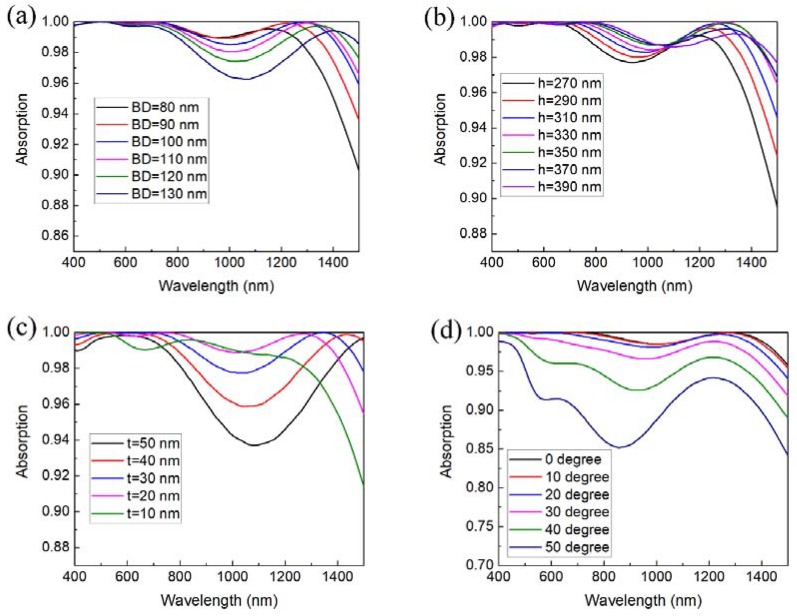
(**a**) Absorption spectra versus base diameter of the top TiN nanocone with the parameters fixed at *h* = 330 nm, and *t* = 25 nm; (**b**) absorption spectra versus height of the top TiN nanocones with *BD* = 100 nm, *t* = 25 nm; (**c**) absorption spectra versus thickness of the Al_2_O_3_ spacer layer with *BD* = 100 nm, *h* = 330 nm; (**d**) absorption spectra versus incident angles.

Figure 4a shows the absorption spectra with respect to different base diameters of the TiN nanocone, while the other parameters are fixed at *h* = 330 nm and *t* = 25 nm. The electric and magnetic fields in the unit cells are strongly influenced by the dimensions of the absorber. With increase of *BD*, the waveband with high absorption is broadened and the absorption peak at the longer wavelength range has a redshift as shown in Figure 4a. One notes that absorption at some wavelengths drop a little due to the mismatch of impedances of the electrical and magnetic resonances. As shown in the spectra, the base diameter has great impact on the absorption spectra, which can be used in tailoring the absorption band. In the waveband of 400–1500 nm, the absorption spectrum with *BD* equal to 100 nm is better than other cases, with broadband high absorption (>98.5%) from 400 nm to 1420 nm.

Meanwhile, the height of the nanocone can also be used in tailoring the absorption performance. With increasing the height of nanocones, antireflection should be strengthened. The average absorbance increases with the height, enhanced by the larger surface area of the nanocones. From the absorption spectra in Figure 4b, the absorption spectrum is broadened with the bigger height, while the absorption at the long-wavelength edge decreases slightly in the studied range, with the redshift of the absorption peak. With the height 330 nm, the absorption exceeds an average of 99.6% from 400 nm to 1500 nm, which is much better than the previously reported absorption with TiN nanodisk arrays, averaging 98.1% from 400 nm to 850 nm [24].

At last, the absorption performance with respect to different thickness of the spacer layer is studied, with the other parameters fixed at *BD* = 100 nm and *h* = 330 nm. By varying the thickness from 10 nm to 50 nm, an obvious redshift of the absorption peak is observed that the absorption band is broadened as shown in Figure 4c. Nevertheless, the impedance mismatch in the middle of the considered range gets larger with the thicker spacer layer, resulting in the decay of the absorption from 800 nm to 1400 nm, which is undesirable for achieving the broadband near-unit absorption. Among these thicknesses, the case of the thickness 25 nm performs the best. In addition, the absorption is found to be polarization-insensitive to the incident light, attributed to the rotational symmetry of the nanocone. As seen in Figure 4d, the average absorption remains about 90% at an oblique incidence up to 50°, which is desirable for solar radiation harvesting and thermal emission applications.

To qualitatively explain the dependence of the absorption spectra on base diameter of the TiN nanocone and thickness of the Al_2_O_3_ spacer layer, the LC model [32] is introduced as shown in Figure 1d. The absorption peak at the longer wavelength range is attributed to the excited magnetic resonance in the spacer layer. When magnetic resonance is excited, eddy currents are induced and flow within a penetration depth from TiN surfaces due to the strong skin effect. The movement of charges or drifting currents cause inductances *L_NC_* at the nanocone surface and *L_sub_* at the Al substrate. Since displacement current is formed between the nanocone and the substrate, a capacitor *C_spacer_* can be reasonably added. Also, a capacitor *C_air_* can be introduced for the reason that displacement current is generated between the adjacent nanocone surfaces. Magnetic resonance occurs at the wavelength *λ*_0_, which zeros the total impedance of the circuit with the following equation:

(1) $$\lambda_{0}=2\pi c_{0}\sqrt{\frac{L_{NC}+L_{sub}}{1/C_{spacer}+1/C_{air}}}$$

The geometric effect on the magnetic resonance wavelengths from spectra as shown in Figure 4 can be understood using the LC model. The capacitance and inductance increase with a larger area in which induced currents exist at the TiN surfaces. Therefore, a larger *BD* would effectively induce current in a larger area, resulting in larger *L_NC_* and *C_spacer_*, then a larger *λ*_0_, which is consistent with the redshift of absorption peak, with larger *BD* observed in Figure 4a. Moreover, the bigger *h* can make adjacent nanocone surface closer, which will cause the increase of *C_air_* and the redshift of the absorption spectra in Figure 4b.

**Figure 5:**
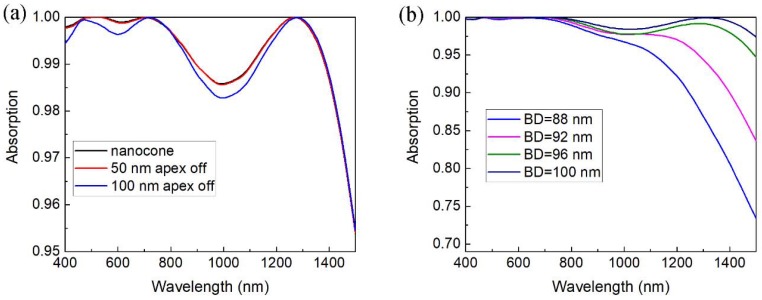
(**a**)Absorption spectra with different height apex-offs when *BD = p =* 100 nm, *t* = 25 nm and *h* = 330 nm; (**b**) absorption spectra with respect to different *BD* while the other parameters are fixed at *p =* 100 nm, *t* = 25 nm and *h* = 330 nm.

In realistic applications, the manufacturing error will be introduced into the MPA structure. As shown in Figure 5, the absorption spectra in cases of apex-off and varying base diameter were simulated. In the apex-off case, the apex of the TiN nanocone is supposed to be cut off as a result that the nanocone becomes prismoid. The absorption spectra say that the apex-off shows little influence on the absorption spectra even to 100 nm apex-off in Figure 5a. As a result, the prismoid structure can substitute the nanocone structure in the fabrication process. In above discussions, the base diameter is set to be equal to the period, though the case of base diameter smaller than period should be clarified. According the absorption spectra shown in Figure 5b, the overall absorption of the MPA gets lower with the decrease of the base diameter, especially, in the long wavelength range. This result can be expected from the reduction of the base area and increase of the separation between the adjacent nanocones.

**Figure 6:**
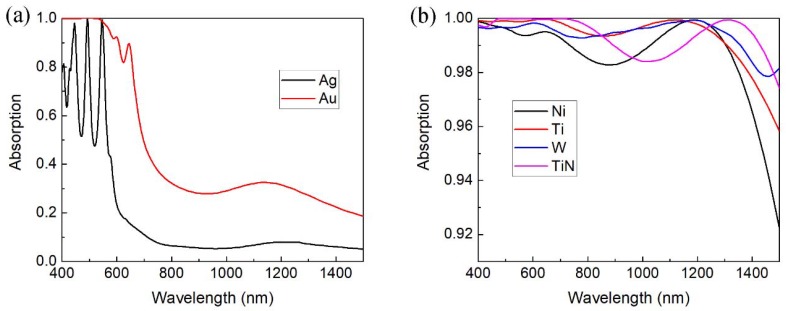
(**a**) Absorption spectra of nanocone MPA based on noble metals gold (Au) and silver (Ag); (**b**) absorption spectra of nanocone MPA with refractory TiN or metals.

Considering other metal counterparts of TiN, the MPA with refractory metal material shows intriguing absorption performance with the same size parameters. The dispersion curves of metals used here are obtained from the Ref. [27]. The MPA based on noble metal with the same size absorbs little incident light in the NIR band as shown in Figure 6a. One can see from Figure 6b that the refractory metals, such as nickel (Ni), titanium (Ti) and tungsten (W), serve as good counterpart of TiN in the nanocone MPA structure with the similar absorption performance. The result indicates that the nanocone MPA structure shows high degree of freedom for choosing different materials.

## 4. Conclusions

In this work, our initially proposed MPA with the periodic circularly shaped TiN nanocone pattern offers a broadband perfect absorption of average 99.6%, from 400 nm to 1500 nm, including a bandwidth with near unit (over 98.5%) absorption from 400 nm to 1420 nm. The realization of the intriguing absorption is on account of the strong LSPR, gap plasmons among the nanocones, and gap plasmons in the gap. The electric resonant absorption is dominant at the shorter wavelength range and the magnetic resonances play more important roles at the longer wavelength range. Their coupling is essentially behind the realization of the broadband absorption. The MPA shows polarization-insensitivity at normal incidence and functions well with good acceptance ability at an oblique incidence of up to 50°. By tailoring the dimensions of the metasurface structure, the perfect absorption band can be tuned according to the practical demand even covering the entire visible-to-NIR range. In addition, the nanocone structure shows large tolerance to apex-off error and small dismatch of base diameter and period. In conclusion, the refractory MPA proposed in this work shows broadband perfect absorption, is promising in light trapping applications and is promising in designing thermophotovoltaic devices.
